# Premarital sexual practices and its predictors among in-school youths of shendi town, west Gojjam zone, North Western Ethiopia

**DOI:** 10.1186/1742-4755-11-49

**Published:** 2014-06-25

**Authors:** Alemayehu Bogale, Assefa Seme

**Affiliations:** 1 College of Health and Medical sciences, Haramaya University, Haramaya, Ethiopia; 2 School of public health, College of Health Sciences, Addis Ababa University, Addis Ababa, Ethiopia

**Keywords:** Youths, Premarital sex, Sexual initiation, Ethiopia

## Abstract

**Background:**

Youth who begin early pre-marital sexual activity are more likely to be engaged in unsafe sex. Early sexual debut puts them at increased risk for acquiring or transmitting sexually transmitted infections, including HIV; and makes them highly vulnerable to unwanted pregnancy and its consequences. This study was conducted to assess premarital sexual practices and its predictors among in-school youths in North West Ethiopia.

**Methods:**

A cross-sectional study was carried among 826 in school youths from December; 2011 to January; 2012 in Shendi town. A multistage sampling technique was used to select the study participants. Binary and multiple logistic regression analyses were performed to examine the relationship between premarital sexual practices and selected exposure variables.

**Results:**

Nearly one fifth 157 (19%) of the participants reported having had premarital sexual intercourse, of which 91 (22.7%) were males and 66 (15.5%) were females. The mean (SD) age at first sexual intercourse was 16 .48 (1.59) for males and 15.89 (1.68) for females. More than three - fourth of sexually active in-school youths engaged in premarital sexual relationship before celebrating their 18th birthday. Being greater than 20 years (AOR = 3.67; 95% CI = 1.98, 6.82), living with friends or relatives (AOR = 2.47; 95% CI = 1.46, 4.16), living alone (without parental control (AOR = 2.51; 95% CI = 1.38, 4.55) and watching pornographic movies (AOR = 1.73; 95% CI = 1.18, 2.53) were found to be significantly associated with premarital sexual practices.

**Conclusion:**

A significant number of in-school youths had started premarital sexual activity that might predispose them to different sexual and reproductive health risks. Therefore, various efforts need to be initiated through school-based information, education, and behavioral change communication, interventions, such as life skills education and negotiation.

## Background

According to the 2007 Ethiopian census, youths aged 15–24 years were more than 15.2 million which contributes to 20.6% of the whole population [1]. These very large and productive groups of the population are frequently exposed to various forms of sexual and reproductive health risks including, sexual coercion, early marriage or sexual debut, female genital cutting, unplanned pregnancies, closely spaced pregnancies, abortion, sexually transmitted infections (STIs), and HIV/AIDS [2,3].

Early sexual debut increases young peoples' risk for infection with HIV and other STIs. Youth who begin early sexual activity are more likely to be exposed to high-risk sex, often having multiple partners and revealed that premarital sexual practices range from 11.8% to 23.2% among in-school youths.

Unprotected sexual activity results not only in exposing youths to STIs, including HIV/AIDS, but also unwanted pregnancy for females, which may cause serious health, social and economic problems. In addition, unwanted pregnancy may lead to school dropout and a failure to complete their education. The situation gets worse for those who are not physically and mentally maturated, such as the youth. Most frequently, unwanted pregnancies also end up with unsafe abortion, which can lead to death and loss of life [2].

According to the 2005 Ethiopian behavioral surveillance survey, the prevalence of premarital sexual practices among in-school youths in Amhara Region was 4.5% [4]. Moreover, different studies conducted afterwards in the region showed that premarital sexual practices among in-school youth are increasing. With the higher level of HIV infection and poorer sexual and reproductive health outcomes among youths [5,6], it is crucial to identify the determinants of sexual activity to inform policy makers and local program managers. However, in-school youth premarital sexual practice and its related health effects were not dealt in-depth within the study area. Besides, most youths in secondary education in Shendi town are living far apart from their families and in an environment away from home without the usual familial control. Therefore, studying premarital sexual practices and its predictors is an essential issue. Thus, this study tried to explore premarital sexual practices and associated factors among in-school youths in Shendi town of North West Ethiopia.

## Methods

### Study design and setting

A cross-sectional study with quantitative and qualitative data collection methods was conducted from December 2011 to January 2012 to assess the prevalence of premarital sex and associated sexual and reproductive health risks among in-school youths of Shendi town, West Gojjam Zone, Amhara National Regional State, Ethiopia. The town is located 427 kilo meters to the North West of Addis Ababa. There are 2 high schools in the town - Dejazemach Bekele high school and Mekonnen Shendi preparatory school.

### Target population

#### Source population

The source population for the study included all in-school youths who were residing in Shendi town or its surrounding kebeles (districts) and who were schooling during the survey.

### Study population

The study population was in-school youths aged 15 to 24 years who were enrolled as a regular day-time student in the 2011/2012 academic calendar.

### Sampling method and data collection

#### Sample size determination

Sample size was calculated using a single population proportion formula based on the assumptions of 20.2% prevalence (P) of premarital sex among in-school youths in Injibara town of Awi zone [5], a 95% confidence level (Za/2), a 4% margin of error (d), a design effect of 2 and a 10% non-response rate. Accordingly, the total sample size calculated was about 851.

### Sampling strategy

A multistage cluster sampling with proportional to size allocation technique was used to select the required number of study subjects as briefly described below. First, thirty four sections were randomly selected out of the total seventy three sections and the samples assigned to each school were distributed proportional to the section size. All never married, day-time; regular students who were aged 15 to 24 years and schooling at the time of the study were identified from the register.

### Data collection

A structured, pre-tested and self-administrated Amharic (a local language) questionnaire was used to collect the required information. Training was given for data collectors and supervisors for 2 days about, the contents of the questionnaire, its administration and issues related to confidentiality of the responses and the rights of the respondents. The data collection was coordinated by the first author and supervised by high school teachers.

To supplement the quantitative findings, four focus group discussions (FGDs) among purposively selected 15–24 yrs in-school youths were conducted. The FGDs, segregated by sex and school, were conducted using semi-structured and open-ended questions which enabled the discussants to reflect on pre-marital sexual practices, predisposing factors and reproductive health risks; by using a pre prepared discussion guide. In addition to notes taken, the discussants' ideas were tape-recorded.

The study used premarital sexual practices as the dependent variable and socio demographic characteristics (age, sex, grade, parental education and occupation), peer pressure, substance use (alcohol, cigarette and khat), religion and living arrangement as independent variables.

### Statistical analysis

The quantitative data were first entered into Epi Info version 3.5.1 and later exported to and analyzed using SPSS version 16.0. Descriptive statistics was computed to determine the frequencies of the dependent and independent variables. Bivariate analyses were done to evaluate associations of each independent variable with the outcome variables. Variables which showed significant association with the outcome variables in the bivariate analyses were entered into multiple logistic regression model to control for confounding and identify independent predictors of premarital sexual practices. Statistical significance was set at a P value of <0.05. The tape-recorded qualitative data were first transcribed, translated and then thematically analyzed. The emerged themes of the qualitative findings were used to supplement the quantitative findings.

### Ethical review

The study was approved by the Institutional Review Board (IRB) of College of Health Sciences at Addis Ababa University. Written permission letter was obtained from all concerned authorities. Verbal consent from each participant was obtained after explaining the purpose of the study. The right of participants to refuse or not to respond to questions they don’t feel comfortable with or discontinue participation at any time was ensured. Confidentiality was kept at each step of the data collection and then after.

## Results

### Socio-demographic characteristics of the study participants

Eight hundred twenty six in-school youth were willing to respond to the questionnaire making a response rate of 97.1%. Of the total respondents, little more than half (51.5%) were females. The majority 732 (88.6%) of the study participants were in the age group of 15–19 years, with a mean (SD) age of 17.6 (±1.4). Also more than half (51.6%) were grade nine students. With regard to ethnic and religious background, eight hundred fifteen (98.7%) were Amharas and 96.7% were followers of Orthodox Christianity, respectively. More than two-third (69%) of the students reported that they attend religious services on a daily basis. With regard to living arrangements, 384 (46.5%) were living with friends or relatives while 165 (20%) reported living alone with out closer familial control (Table 1).

**Table 1:** Socio-demographic characteristics of in-school youths in Shendi town, West Gojjam, January 2012

**Variables**	**Frequency (n = 826)**	**Percent**	
Sex			
Male	401	48.5	
Female	425	51.5	
Age (years)			
15-19	732	88.6	
20-24	94	11.4	
Grade level			
9th	426	51.6	
10th	288	34.9	
11th	60	7.3	
12th	52	6.3	
Ethnicity			
Amhara	815	98.7	
Others	11	1.3	
Religion			
Orthodox Christian	799	96.7	
Muslim	17	2.1	
Protestant	10	1.2	
How often do you attend religious services?			
Daily	569	68.9	
Once in a week	219	26.5	
Once in a month	29	3.5	
Once in a year	9	1.1	
Respondent live with			
Friends or relatives	384	46.5	
Both biological parents	221	26.8	
Single biological parent	56	6.8	
Alone	165	20.0	

Majority 653 (79%) of students' parents were currently married and reside in rural areas; 694 (84%). Four hundred fifty eight (58.2%) of the students had illiterate mothers and more than a quarter 197 (26.5%) had illiterate fathers. The majority 646 (86.8%) of the students fathers, were farmers while 418 (53%) and 323 (41%) of the students’ mothers were farmers and housewives, respectively. Majority 610 (73.8%) of the students perceived that they are from families with medium economic status (Table 2).

**Table 2:** In-school youth parents’ socio-demographic characteristics in Shendi town, West Gojjam zone, January 2012

**Variables**	**Frequency**	**Percent**	
Parents’ current marital status			
Currently married	653	79.1	
Divorced	108	13.1	
Widowed	65	7.9	
Parents’ place of residence			
Urban	132	16.0	
Rural	694	84.0	
Mother’s educational status			
Illiterate	458	58.2	
Read and write	234	29.7	
Primary school [1-8]	71	9.0	
Secondary school [9-12]	13	1.7	
Higher education	11	1.4	
Father’s educational status			
Illiterate	197	26.5	
Read and write	361	48.5	
Primary school [1-8]	140	18.8	
Secondary school [9-12]	28	3.8	
Higher education	18	2.4	
Father’s occupation			
Farmer	646	86.8	
Merchant	58	7.8	
Gov’t/private employ	33	4.4	
Others	7	1.0	
Mother’s occupation			
House wife	323	41.0	
Farmer	418	53.1	
Merchant	30	3.8	
Others	16	2.1	
Perceived economic status of parents			
Poor	137	16.6	
Medium	610	73.8	
Rich	79	9.6	

### Substance use by in-school youths

Five hundred eighty eight (71.2%) students reported consumption of local alcoholic drinks (local beer, also called '*tela,*' and/or' *areke*') while only eight (1%) reported consumption of “khat” at least once in their life time. Of those who consumed alcohol, 393 (66.8%) had drunk occasionally, 170 (28.9%) once or twice a week and 25 (4.3%) daily. Only one student reported smoking cigarette on a daily basis.

### Sexual behavior and condom use by in-school youths

Little more than half 426 (51.6%) of the study participants reported to have ever seen pornographic movies or read some pornographic magazines, while nearly one-third 257 (31%) of the students reported having had boy/girlfriends. Of the total study participants, 157 (19%) reported to have had premarital sexual intercourse at the time of the survey of which 66 (42%) were females. The mean age (±SD) at first sexual intercourse was 16.5 (±1.6) for males and 15.9 (±1.7) for females. Out of all sexually active youths, 20 (12.7%) had their first sexual intercourse before the age of 15 years.

**Figure 1:**
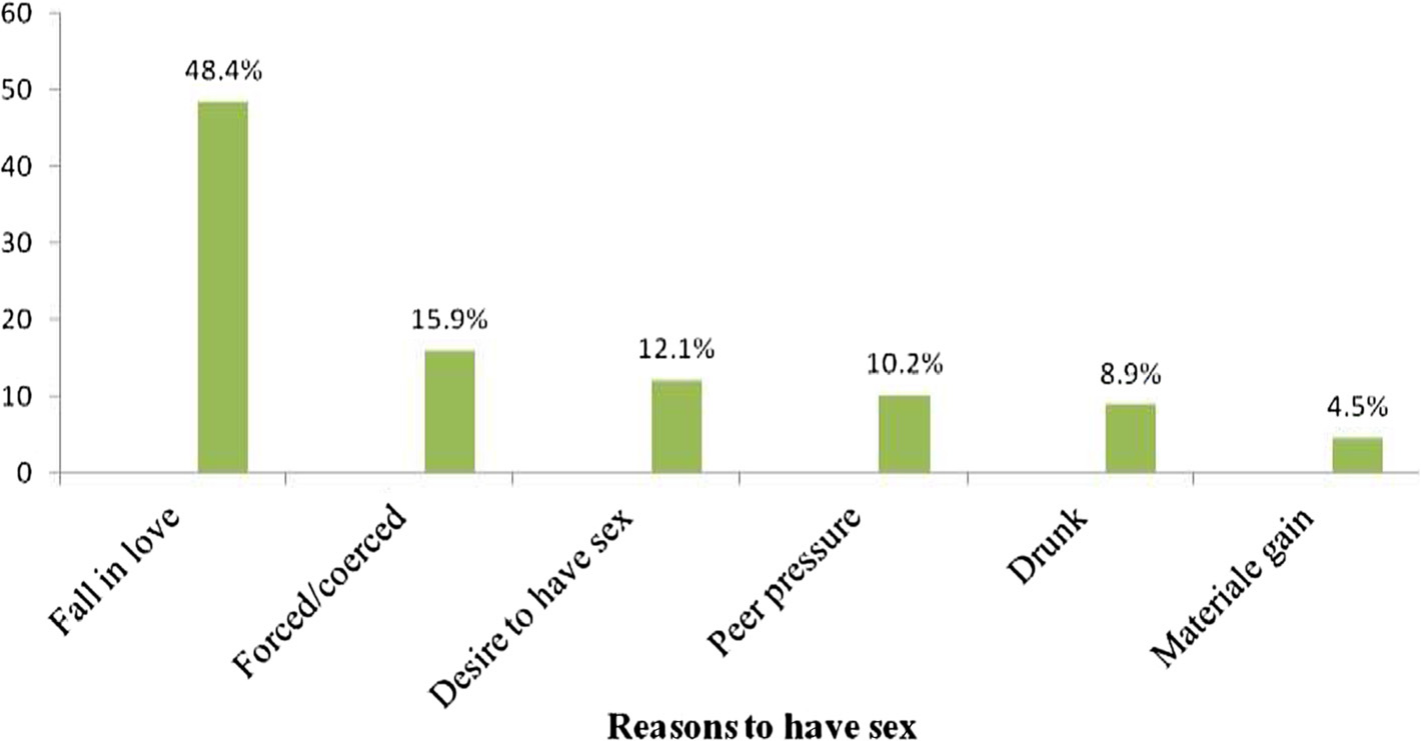
Reasons to have sex among in-school youth in Shendi town, West Gojjam Zone, January 2012.

Nearly half 76 (48.6%) of the sexually experienced students reported that their first sexual partner was a boy/girlfriend outside the school, 64 (40.8%) experienced sex with a school boy or girl friend and 12 (7.6%) had sexual experience with commercial sex workers. Seventy three (46.5%) of sexually experienced students reported that their first sexual partner was of the same age while 55 (35%) and 25 (15.9%) had first sexual encounter with older and younger age partners, respectively. When asked on the number of life time sexual partners they ever had, most 118 (75.2%) sexually active students reported that they had only one sexual partner while 39 (24.8%) reported having had two or more sexual partners (Table 3). Almost half (48.4%) of youths claimed that the main reason for the initiation of first sexual intercourse was falling in love (Figure 1).

**Table 3:** Sexual behavior of in-school youths in Shendi town, West Gojjam Zone, January 2012

**Variables**	**Frequency n = 826**	**Percent**	
Exposure to pornographic materials			
Yes	426	51.6	
No	400	48.4	
Had boy/girl friends			
Yes	257	31.1	
No	569	68.9	
Ever had sexual intercourse			
Yes	157	19.0	
No	669	81.0	
Age at first sexual intercourse (n = 157)			
Less than 15 years	20	12.8	
15-18 years	128	81.5	
Greater than 18 years	9	5.7	
First sexual partner			
School boy/girl friend	60	40.8	
Out of school boy/girl friend	76	48.4	
Commercial sex workers	12	7.6	
Relatives	5	3.2	
How old was the first sexual partner			
Younger than the respondent	25	15.9	
Same age as the respondent	73	46.5	
Older than the respondent	55	35.0	
Not known	4	2.6	
Main reasons for first-time sex			
Fall in love	74	48.4	
Forced/coerced	25	15.9	
Desire to have sex	19	12.1	
Peer pressure	16	10.2	
Drunk	14	8.9	
Material gain	7	4.5	
Number of sexual partners			
One	118	75.2	
Two	31	19.7	
Three and above	8	5.1	

About three in ten (28.7%) sexually active in-school youths had used condom during their first sexual intercourse. Eighty-eight (56%) claimed that they had used condom in the last 12 months before the survey. One third 30 (34.1%) of respondents reported using condom consistently for the last 12 months. Reasons for none or inconsistent condom uses were cited as follows: trusting partner 49 (38.6%), ashamed to ask partner 31 (24.4%), fear to buy from shops or pharmacies 21 (16.5%), lack of interest 12 (9.4%), lack of knowledge to use 10 (7.9%) and thought it reduces sexual pleasure 8 (6.3%). Only 8 (5.1%) of sexually active in-school youths reported signs and symptoms of sexually transmitted infections.

### Factors associated with premarital sexual practices and condom use

There was a positive association between age, living arrangement and watching pornographic movies with ever having premarital sex. Youths aged 20 years or more were nearly four times more likely to experience premarital sex compared to younger ones (AOR = 3.79, 95%CI = 2.04,7.02). Youths living with their friends or relatives were more than two times more likely to experience premarital sex compared to youths living with both biological parents (AOR = 2.53, 95% CI = 1.50, 4.29). Similarly in-school youths who reported to live alone were more than two times more likely to exercise premarital sex compared to youths living with both biological parents (AOR = 2.62, 95% CI = 1.44, 4.77). The study also showed that youths who reported watching pornographic movies were about two times more likely to experience premarital sex than those who didn't (AOR = 1.74, 95% CI = 1.19, 2.54) (Table 4).

**Table 4:** Relationship between socio-demographic variables and sexual behavior among in-school youths in Shendi town, West Gojjam Zone, January 2012

**Variables**	**Ever had premarital sex OR (95%)CI**			
	**Yes**	**No**	**Crude**	**Adjusted**
**Sex**				
Male	91	310	1.59(1.12, 2.26)**	1.13(0.77, 1.67)
Female	66	359	1	1
**Age**				
15-19	121	611	1	1
20-24	36	58	3.13(1.98, 4.96)**	3.79(2.04,7.02)**
**Educational level**				
9-10	132	582	1	1
11-12	25	87	1.26(0.78, 2.05)	0.58(0.33, 1.05)
**Currently living with**				
Both biological parents	21	200	1	1
Live with single parent	11	45	2.32(1.04, 5.17)**	2.01(0.87, 4.61)
Live with relatives or friends	83	301	2.62(1.57, 4.37)**	2.53(1.50, 4.29)**
Live with alone	42	123	3.25(1.83, 5.75)**	2.62(1.44, 4.77)**
**Alcohol drinking**				
No	31	207	1	1
Yes	126	462	1.82(1.19, 2.78)**	1.54(0.98, 2.41)
**Perceived economic status of the**				
**family**				
Poor	37	100	1	1
Medium	106	504	1.45(0.89,2.35)	0.70(0.44, 1.11)
Rich	14	65	1.25(0.67,2.34)	0.77(0.37, 1.59)
**Watching pornographic movies**				
No	56	344	1	1
Yes	101	325	1.90(1.33, 2.73)*	1.74(1.19, 2.54)**

NB ** = statistically significant association.

The multivariate logistic regression analysis also showed that condom use was associated with sex, discussion on sexuality with their close friends and perceived economic status of the family. Males were more than two times more likely to use condom as compared to females (AOR = 2.56, 95% CI = 1.26, 5.22), youths who discuss on sexuality issues with their close friends were nearly three times more likely to use condom than those who didn't discuss (AOR = 2.84, 95% CI = 1.27, 6.37). On the other hand, youths whose perceived family economic status was medium were found to be three times more likely to use condom as compared to those with perceived poor economic status of the family (AOR = 2.92, 95% CI = 1.28, 6.68) (Table 5).

**Table 5:** Variables evaluated for possible association of condom use by in-school youths of Shendi town, West Gojjam Zone, January 2012

**Variables**	**Condom use OR (95%)CI**			
	**Yes**	**No**	**Crude**	**Adjusted**
**Sex**				
Male	59	32	2.35(1.22, 4.50)**	2.56(1.26, 5.22)**
Female	29	37	1	1
**Age**				
15-19	66	55	1	1
20-24	22	14	1.31(0.61, 2.79)	1.61(0.60, 4.29)
**Educational level**				
9-10	76	56	1	1
11-12	12	13	0.68(0.28, 1.60)	0.57(0.19, 1.67)
**Currently living with**				
Both biological parents	12	9	1	1
Live with single parent	6	5	0.90(0.20, 3.90)	1.02(0.20, 5.05)
Live with relatives or friends	45	38	0.88(0.33, 2.33)	1.08(0.36, 3.20)
Live with alone	25	17	1.10(0.38, 3.18)	1.21(0.36, 4.09)
**Alcohol drinking**				
No	20	11	1	1
Yes	68	58	0.64(0.28, 1.45)	0.47(0.18, 1.19)
**Discussion with their close**				
**friends**				
No	17	24	1	1
Yes	71	45	2.22(1.07, 4.59)**	2.84(1.27, 6.37)**
**Perceived economic status of the family**				
Poor	15	22	1	1
Medium	68	38	0.57(0.24,1.35)	2.92(1.28, 6.68)**
Rich	5	9	0.50(0.16, 1.54)	0.77(0.20, 2.98)

NB ** = statistically significant association.

### Results of the qualitative data collection

A total of 32 participants were involved in four focus group discussions. The themes emerged from the discussion are presented as follows:

### Youth’s premarital sexual practices and its consequences

According to the discussants; youth’s premarital sex is becoming a common practice in the study area and considered by youths as a fashion. ‘*Let alone in secondary schools where one can find older youths, now a days it is becoming a usual practice in primary schools as well*’. Most of the students are coming from rural areas and renting a house alone or with their friends without closer monitoring or supervision from parents/or guardians. Such exposure to new environment and obtaining a relative freedom (being away from parental monitoring and control) makes youths to be highly vulnerable to such early sexual practices. One of the discussants said the following,

“*Now a days, most of the students coming from rural areas have more than one boy/girl friends just for sex*”. He added, “*Surprisingly*, *my intimate male friend, 10*^*th *^*grader, had sexual experience with more than 10 sexual partners in this school*”.

Most students recognize problems associated with premarital sexual practices but they are involved on it. They just practice it because they observe others practicing it. One of the female discussants said,

“*Since sexual practices among in-school youth is well recognized in the community, most of the landlords do not want to rent their house to female students due to fear of conflicts between male visitors/strangers who visit the female tenant”*

### Early sexual initiation and multiple sexual partners

Majority of the focus group discussants agreed that early sexual initiation and having multiple sexual partners is a common phenomenon in the study area. They emphasized that early and premarital sexual practices are the basis for STIs and HIV/AIDS transmission, unwanted pregnancy, abortion, school dropout and premature death. An 11th grade female student sadly explained how she lost her closest friend:-

“….*due to premarital and unsafe sexual practices in early age, one of my closest friend had lost her life ……after unprotected sex she became pregnant… she died when she tried to abort a five months old fetus using herbs from a traditional healer”*.

### Condom use

Majority of the FGD discussants didn’t consider the use of condom as an acceptable means of prevention because of perceived reduction in sexual pleasure. Youth discussants raised the flesh-to-flesh contact as the most satisfying part of the sexual intercourse. According to these participants, some of the reasons for non-use of condom are perceived reduction in the sexual pleasure.

“Using *condom is like walking in the rain with a person enclosed by plastic sheet…., you will never feel the rain…it means you never taste the rain.”* a young male student discussed.

Others have cited lack of decision making power as reasons for non-use. A 17 years old girl discussant said *“…The decision for condom use is made by males, we females can’t decide and this makes females not to use condom”*.

## Discussions

In this larger study conducted with the aim of assessing the magnitude of premarital sex and its predictors among in-school youths. About one-fifth (19%) of high school and preparatory school students reported to have had premarital sexual intercourse. The result is more consistent with a study done in Ambo [7]. However, it is higher than previous study results from the Ethiopian Behavioral Surveillance Survey, Bullen Woreda of Benishangul Gumuz region of Ethiopia and Malaysian school survey [4,8,9], and lower than the study findings of Nekemte [10], West Gojjam [6] and Nepal [11]. This discrepancy may be due to the difference in socio– cultural and schooling status among the study participants.

With regard to premarital sexual practice, more than 75% of in-school youths reported that they had started practicing sex before celebrating their 18th birthday. In addition, early sexual practice initiators were more likely to be involved in subsequent high risk sexual behaviors such as having multiple sexual partners and no or inconsistent condom use. This finding is in line with several other studies previously done in diverse settings [4-6,10,12]. Moreover, female students sexual partners’ age at sexual debut were more likely to be older, who might have experienced different sexual and reproductive health problems. This early initiation of sexual activity and having an older age sexual partner, particularly among female students, may prolong the period of exposure to risks of unwanted pregnancy and contracting STIs, including HIV, during their reproductive life span.

The results of this study showed that, among nonsexual risky behaviors, like viewing pornographic materials at earlier age was an independent predictor of premarital sexual initiation. This finding was in agreement with the study conducted in North East Ethiopia [12]. Moreover, premarital sexual activity was found to be significantly more common among youths coming from rural areas compared with in-school youth living in a relatively urban area. The result is consistent with earlier study findings conducted in Nekemte and Tanzania [10,13]. The most likely explanation is due to a difference in living arrangement, i.e. youth coming from rural areas often live with relatives instead of their biological parents, and thus lack familial control, and lack of adequate knowledge about sexual and reproductive health risks.

One in four students reported having had two or more sexual partners in their life time. This finding was comparable with the results of the Ethiopian Behavioral Surveillance Survey (EBSS) 2005 [4]. However, it was lower than the finding in Nekemte [10] but higher than the findings in Injibara and Gedeo zones of Ethiopia [5,14]. One alarming finding in this particular study is that a higher proportion of the sexually active male students (13.2%) had sexual contact with commercial sex workers. This finding was consistent with the study findings conducted in Nekemte but more than two times higher than the study findings conducted in West Gojjam zone [10,6].

Forced sex was most commonly reported in the Amhara region (14.9%) [4]. This particular study was also witnessed that nearly one in six (15.9%) of female students reported forced sexual initiation. Performing premarital sexual practices are statistically significant with age, living arrangement, and watching pornographic movies.

Another typical feature that makes youth sexual activity risky is the absence or incorrect use of condom during sex. Accordingly, only 28.7% of the sexually active youth reported using a condom during their first sexual practice, and only 34.1% reported that they used condom consistently during the past 12 months prior to the study. The major reason mentioned for inconsistent or none use of condom was; trusted their sexual partner (38.6%). This finding is consistent with the results of the 2005 Ethiopian Behavioral Surveillance Survey (EBSS) [4]. However, it is higher than previous study results conducted in Ambo [7] and lower than the study findings of Thailand [15]. Of those male students who practiced sex with commercial sex workers, only 26.7% reported that they had used condom correctly and consistently. The main reason for none or inconsistent use was fear of buying from shops or the pharmacy. The reported low utilization rate of condom in this study may indicate the prevailing fact that high-risk sexual behaviors are still widely prevalent among in-school youth unlike what it is thought to be.

Results of the FGDs support that condom utilization by youths are minimal. One young boy discussant explained the situation as follows, “using *condom is like walking in the rain with a person enclosed by plastic sheet…., you will never feel the rain…it means you never taste the rain.”* Another female discussant also said, “*In our culture, men are dominant over women in all matters associated with sex and sexual relationship. As a result, the decision to use condom is often made by men, and this makes women not to use condom despite they may get access to it*”.

### Limitations of the study

Since this study touches very sensitive and very personal issue; social desirability responding cannot be ruled out. Also, the cross-sectional nature of the study makes it impossible to draw inferences about the direction of relationship between the dependent and independent variables. Moreover, the study is retrospective and thus is subjected to recall bias.

## Conclusion

In conclusion, this particular study indicated that a substantial proportion of in-school youths were practicing premarital sexual practices. Being greater than 20 years, students living arrangement (i.e. living with their relatives, friends or alone), and watching pornographic movies were found to be the significant and independent predictors of premarital sexual practice. Also, a significant number of in-school youths had started sexual intercourse very early and are involved with high-risk sexual practices, including multiple sexual partner, unprotected sex and sex with commercial sex workers. Therefore, health care authorities in the different hierarchy should give attention to the identified problems in order to promote the sexual and reproductive health of in-school youth.

## Competing interest

The authors declare that they have no competing interest.

## Authors’ contributions

AB, and AS: participated in all steps of the study from its inception to write up. They have reviewed and approved the submission of the manuscript. Both authors read and approved the final manuscript.
